# Modeling Infrared Signal Reflections to Characterize Indoor Multipath Propagation

**DOI:** 10.3390/s17040847

**Published:** 2017-04-13

**Authors:** Álvaro De-La-Llana-Calvo, José Luis Lázaro-Galilea, Alfredo Gardel-Vicente, David Rodríguez-Navarro, Ignacio Bravo-Muñoz, Georgios Tsirigotis, Juan Iglesias-Miguel

**Affiliations:** 1 Department of electronics, University of Alcalá, Alcalá de Henares, 28801 Madrid, Spain; alvaro.llana@uah.es (Á.D.-L.-L.-L-C.); alfredo.gardel@uah.es (A.G.-V.); david.rodriguezn@edu.uah.es (D.R.-N.); ignacio.bravo@uah.es (I.B.-M.); juan.iglesiasm@edu.uah.es (J.I.-M.); 2 Informatics Engineering Department, Eastern Macedonia and Thrace Institute of Technology, 65404 Kavala, Greece; tsirigo@teiemt.gr

**Keywords:** reflection model, IR signals, multipath propagation, local positioning systems

## Abstract

In this paper, we propose a model to characterize Infrared (IR) signal reflections on any kind of surface material, together with a simplified procedure to compute the model parameters. The model works within the framework of Local Positioning Systems (LPS) based on IR signals (IR-LPS) to evaluate the behavior of transmitted signal Multipaths (MP), which are the main cause of error in IR-LPS, and makes several contributions to mitigation methods. Current methods are based on physics, optics, geometry and empirical methods, but these do not meet our requirements because of the need to apply several different restrictions and employ complex tools. We propose a simplified model based on only two reflection components, together with a method for determining the model parameters based on 12 empirical measurements that are easily performed in the real environment where the IR-LPS is being applied. Our experimental results show that the model provides a comprehensive solution to the real behavior of IR MP, yielding small errors when comparing real and modeled data (the mean error ranges from 1% to 4% depending on the environment surface materials). Other state-of-the-art methods yielded mean errors ranging from 15% to 40% in test measurements.

## 1. Introduction

In recent years, the applications and uses of Global Navigation Satellite Systems (GNSS) have been successfully consolidated. In outdoor environments, they can provide a location with an accuracy of a few meters and sometimes even centimeters if advanced techniques are employed, with unprecedented coverage compared to any other kind of positioning system. This coverage is provided by a large constellation of dedicated satellites. GNSS systems are distributed around the globe and support multiple applications.

A natural development of outdoor positioning would be to transpose the techniques to indoor environments, but GNSS present several problems. The most important of these include the lack of accuracy in Local Positioning Systems (LPS) based on satellite signals, up to a few meters in the best case. Thus, the positioning accuracy of LPS is insufficient for many indoor environment applications. The main reasons are signal attenuation and Multipath (MP) problems in closed areas. Consequently, alternatives are being developed for LPS environments where GNSS methods are not suitable. Several studies have been conducted on LPS based on cameras [1], radio-frequency (such as UWB [2,3], radar [4,5,6], ad hoc networks, GSM or WiFi [7]), voice [8], ultrasound [9] and IR signals [10].

Note that none of these techniques provides a complete solution for LPS, so the use of one or another technique will depend on the requirements of the application in question. The most common method employed to estimate the LPS position of a mobile robot is trilateration, which means using measurements, such as Time of Arrival/Time of Flight (ToA/ToF) [11,12], Time Differential of Arrival (TDoA), Phase Differential of Arrival (PDoA) [10] or Received Signal Strength (RSS) [13], and triangulation techniques, such as Angle of Arrival (AoA) [14].

In indoor environments, IR signals reach the receiver along the Line of Sight (LoS) path, but also along other MPs due to signal reflections on surfaces in the environment. Each of these components reaches the receiver with a different power strength and phase than signals that have traveled along the LoS. When using features of the received signal, such as ToA or PDoA, to detect the emitter or receiver position, the effects of the MP components can reduce the accuracy of the LPS system to the extent that the measurement can become unusable. The number of MP components to be considered is in general unknown and depends on the signal path geometry, signal features, diffraction effects and surface reflections. In addition, the emitter and/or receiver will be moving, further complicating the reduction of MP effects. Accuracy is most severely affected by MPs with similar delays to the LoS, because correlation procedures do not correctly discriminate between LoS and MP components.

Several studies on IR-LPS published by the Electronic Engineering Applied to Intelligent Spaces and Transport Group (GEINTRA) [15], such as [14,16], have been based on recovering the composite signal to be received by the LPS receiver, analyzing and simulating MP effects and developing algorithms capable of correctly discriminating between the LoS and other MPs. In this case, it is necessary to know the model for an IR signal reflection on the different surface materials present in the environment and to obtain the composition (signal power and phase) of each path to the detector from the ray geometry and reflections of each MP. Once this information has been obtained, it is possible to compute the impulse response to the signal channel and analyze the signal MP effects.

Nowadays, Visible Light Communication (VLC) is experiencing a growing interest. In this sense, the use of LEDs with modulated signals can be applied not only for data communication, but also for the development of indoor positioning systems, a goal that is not completely resolved and currently of great interest; the proposed model can be applied to characterize the multipath behavior of optical signals in applications such as indoor positioning and VLC communications.

In fact, there is a current work in-progress based on this reflection model, which computes off-line the estimated behavior of the multipath rays considering the room geometry, and objects are known, so as to try to later mitigate the multipath effects in real time and improve the positioning obtained by a Infrared Position Sensitive Device based system (a simulation tool to perform these calculations automatically from a known room geometry is being developed). Additionally, we are currently working on indoor positioning replacing the ad hoc IREDs with LED lamps for lighting yet installed in the building, just as it would be for the VLC case. By extension, our model could be used in VLC to analyze the multichannel effects of LED lighting systems and to be able to evaluate and quantify the possible distortions and other adverse effects that they generate in the communications.

Therefore, our goal in this study was to develop a reflection model capable of characterizing an environment by modeling any surface material, using a method that obtains the model parameters easily and rapidly without requiring a complex setup.

The paper is organized as follows. First, we provide an overview of previous research on light reflection models. Then, we propose the theoretical model. Next, we describe a procedure to obtain its parameters. Then, we analyze the goodness of fit of the model results using real measurements. After that, we propose a method for obtaining the model parameters. Finally, we report the results of implementing the reflection model, computing the errors in the same way as in [17], and present our main conclusions.

## 2. Background

**Figure 1:**
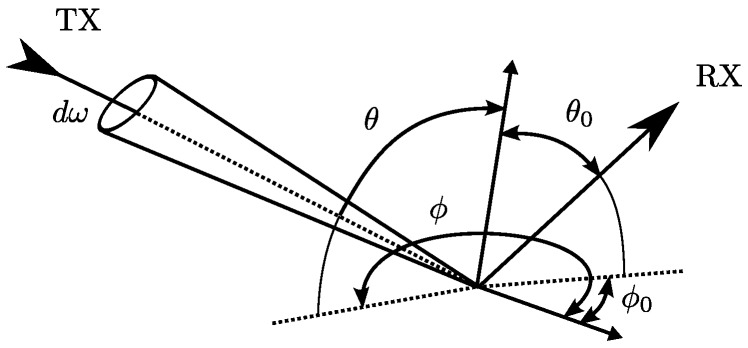
BRDF function variables.

In order to study the reflection of different materials and propose a model suitable for an IR-LPS, we will review the model based on the Bidirectional Reflectance Distribution Function (BRDF). The BRDF is a four-variable function $f\left(\theta ,\phi ,\theta_{0},\phi_{0}\right)$ that describes how light is reflected on an opaque surface. Figure 1 shows the four angles featured in BRDF.

There are several analytical models for the BRDF, and they can be divided into empirical, physical and geometrical models.

**Figure 2:**
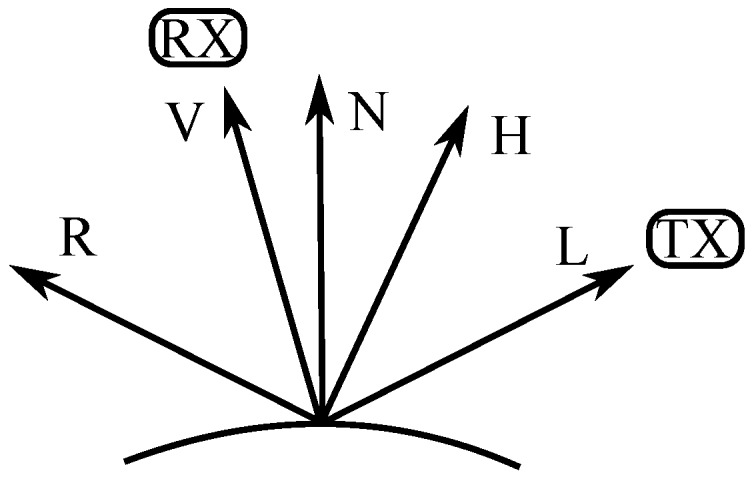
Vectors used on the empirical models for surface light reflection.

Empirical models: Figure 2 presents the vectors used in empirical models, where vectors L, V and R are the ray’s incidence, reception and maximum reflections (according to Snell’s law), respectively, and vector N is the vector normal to the surface.

In [18], a Phong model is introduced, which is an empirical model of light-surface reflection considered as a combination of several types of reflection: diffuse, specular and ambient (surface illumination when not directly illuminated with a light ray). The ambient reflection component is constant; the diffuse component is modeled as a Lambertian surface that depends on the cosine of the angle formed by the vectors $\vec{L}$ and $\vec{N}$; and the specular component depends on the cosine of the angle formed by the vectors $\vec{R}$ and $\vec{V}$.

In [19], the Phong model is slightly modified to include an algorithm with physical restrictions on the light-surface reflection. This model generates a more realistic result, although not as perfect as the diffuse reflection introduced with the Lambertian models or the one modeled by the Phong algorithm. The authors state that both specular illumination intensity and position change with the light orientation of the incident ray. In [20], the Lafortune model is described, a generalization of the Phong model given in [18]. It considers the light reflection as the sum of different components computed with the generalized cosine lobe model described in the same study.

Physical optic models: In [21], a model based on electromagnetic waves is presented. The model is only valid for surfaces that meet certain restrictions; for example, surfaces must be electrical conductors. The model consists of two components: the specular-spike component, which depends on frequency, and the specular-lobe component, which takes into account the dispersion of light reflected on rough surfaces. In [22], another model based on electromagnetic waves is presented. This model divides light reflection into three different components: ideally specular, orientation-diffusion and ideal-diffusion. The specular component and the orientation-diffusion component are obtained from the very first reflection of the light ray. The model depends on the wavelength of the incident light, roughness, angle of incidence and surface refraction index.

In [23], the Oren–Nayar model is described. This is a physical model of light reflection that considers that the surfaces are formed of microfacets with a Lambertian behavior. The microfacets are distributed with a uniform probable density function along the surface, and different undesirable effects may appear as maskings, shadows or inter-reflections.

In [24], a physically-based reflectance model is presented, which combines reflection and diffraction physical phenomena.

Geometrical optic models: In [25], the Cook and Torrance model is presented, which is a modification of the Blinn and Torrance–Sparrow models described previously in [26], and considers that the surfaces are formed of multiple microfacets, and each of the microfacets is considered a perfect specular surface (mirror). The model uses three terms: Fresnel coefficients, microfacet orientation distribution and masking-shadow effects. It employs a distribution function that represents the microfacet distribution on top of the surface material with normal vectors perpendicularly oriented to the surface. This introduces a Fresnel term for specular reflection that depends both on the angle of incidence and the wavelength of the reflected light.

It is important to note that prior to using physical and geometrical models, the parameter values of the surface materials must be obtained empirically, and the properties and characteristics of the surface materials must therefore be measured with high-performance measurement devices, such as gonioreflectometers [27]. For example, with some methods, it is necessary to measure the standard deviation of the surface material roughness, and thus, in some cases, a measurement tool with micrometer accuracy must be used. Moreover, the refraction index of the materials is also required, which is very difficult to obtain for such common building materials as terrazzo, brick walls or plasterboards.

In [17], an analysis is conducted of the most important BRDF models, including those mentioned earlier, and their performance is compared with data obtained with a gonioreflectometer for materials ranging from metals (i.e., chromium) to paints, revealing the reduced accuracy obtained with some of them for different angles of incidence (errors of between 15% and 40%), which will be discussed further in the Results section.

There are several databases containing the reflection model parameters for different materials, but normally, these are only available when considering visible light rays. Consequently, and given the fact that current reflection models do not meet the requirements for our application, here we propose a reflection model suitable for any kind of surface material together with a simplified method for obtaining its parameters.

## 3. Theoretical Framework for an LPS Reflection Model

This section starts from the equation of the optical power received at a detector considered as a point detector located somewhere in the space. Subsequently, the proposed reflection model will be gradually adjusted and refined until attaining the final reflection model.

### 3.1. Initial Considerations

In this section, we describe a model that considers the propagation of an electromagnetic (light) signal inside an ideal channel between the emitter and detector, with a continuous stable and known emitted power and only receiving the LOS component.

**Figure 3:**
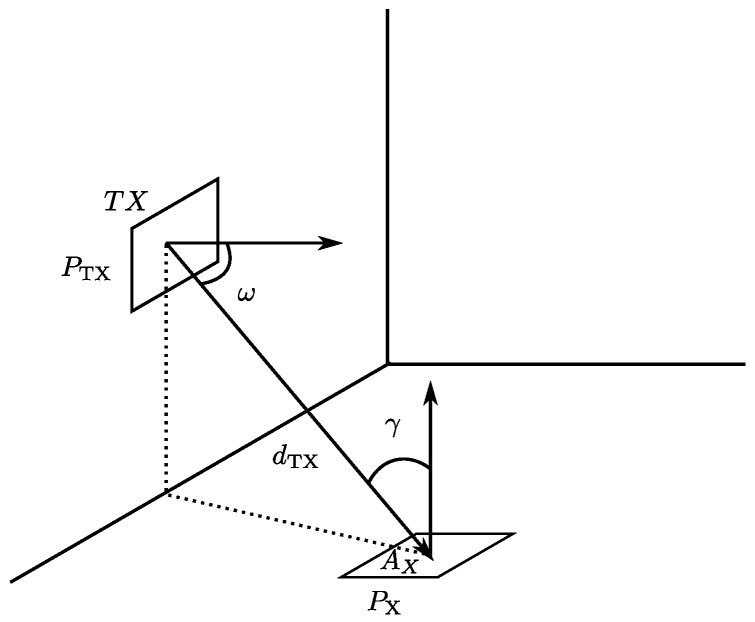
Diagram with the power terms received at detector location *x* ($P_{x}$).

In general, the ratio between the power ($P_{x}$) of the signal received in the detector at a given point in space and the energy emitted from the emitter location (Figure 3) is given by Equation (1):

(1) $$P_{x}=I\left(\omega \right)\frac{1}{d_{\mathrm{TX}}^{2}}F\left(\gamma \right)R\left(\gamma \right)A_{x}=E\left(\omega \right)F\left(\gamma \right)R\left(\gamma \right)A_{x}$$

where $I\left(\omega \right)$ represents the emission pattern, $d_{\mathrm{TX}}$ the LOS distance from emitter to receiver, $F\left(\gamma \right)$ the transmission function of a filter placed in the detector, $R\left(\gamma \right)$ the response of the receiver (including the gain of any optic concentrator and its response) and $A_{x}$ is the effective area of the receiver. $E\left(\omega \right)$ represents the energy per unit area that the emitter generates in the location where the detector is placed.

The emission pattern, $I\left(\omega \right)$, may be expressed as Equation (2):

(2) $$I\left(\omega \right)=\frac{n+1}{2\pi }P_{\mathrm{TX}}\cos^{n}\left(\omega \right)$$

where *n* is the radiation lobe index, $P_{\mathrm{TX}}$ is the emitter power and ω is the angle at which the radiated intensity from the emitter is evaluated with respect to the axial angle of the emitter.

The index *n* is given by Expression (3):

(3) $$n=-\frac{\ln\left(2\right)}{\ln\left(\cos\left(\phi_{1/2}\right)\right)}$$

where $\phi_{1/2}$ is the angle at which the power is half that of the power at $0^{\circ }$.

The *n* index provides information about the directionality of the emitter. High *n* index values represent very directional behavior (narrow emission lobes), whereas low *n* index values represent wide emission lobes, dividing the emitted power into a cone with a higher aperture.

**Figure 4:**
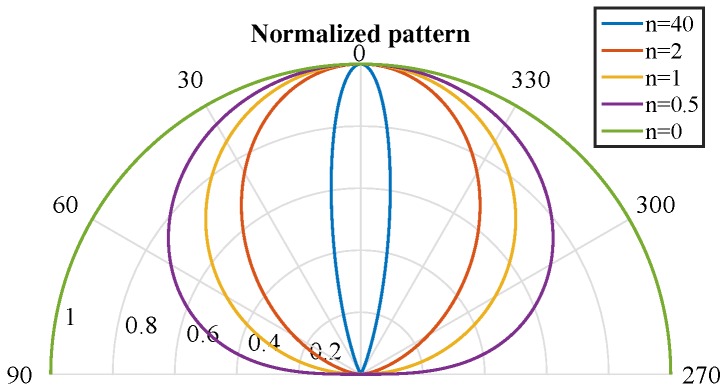
Examples of normalized radiation emission diagrams for different *n* index values.

Figure 4 shows several normalized emission diagrams for an emitter with different values for its *n* index. Note that for n=0, the behavior of the emission is isotropic, and the power emitted is distributed in all directions.

For an *n* index value equal to one, the emission pattern has a Lambertian behavior, with a half-power angle $\phi_{1/2}=60^{\circ }$. As previously described, the higher the *n* index, the narrower the emission.

If the detector does not have a coupled concentrator (optics), its behavior with respect to the axial axis is considered a Lambertian one with n=1 as show in Equation (4):

(4) $$R\left(\gamma \right)=\cos\left(\gamma \right)$$

In this case, without coupling filters, the received signal power will be given by the Lambert expression of Equation (5):

(5) $$P_{x}=\frac{n_{\mathrm{TX}}+1}{2\pi }\frac{1}{d_{\mathrm{TX}}^{2}}\cos^{n_{\mathrm{TX}}}\left(\omega \right)A_{x}\cos\left(\gamma \right)P_{\mathrm{TX}}$$

From the Lambert Equation (5), we can obtain the power signal received by the detector (Rx) along the LOS path from the emitter (Tx). However, as noted earlier, it is not only the LOS signal that will arrive at the detector: other MP components will also impact on the detector.

**Figure 5:**
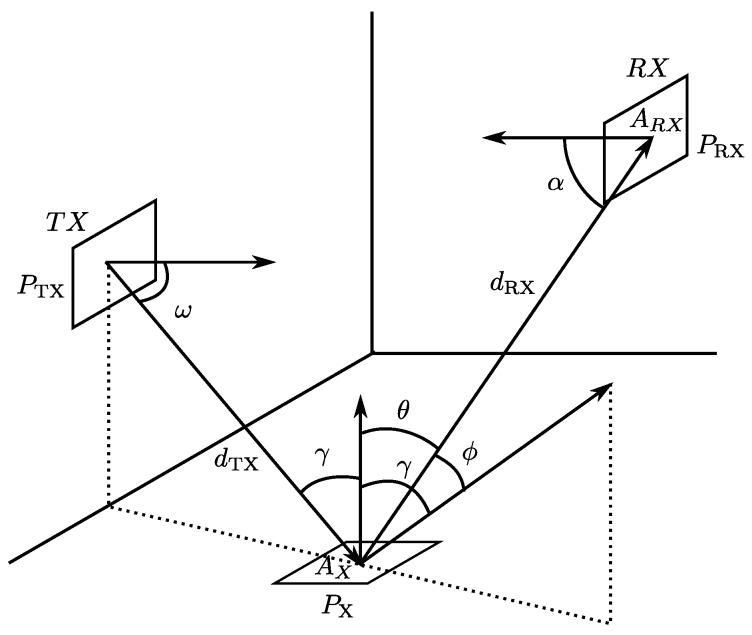
Signal power received from a Multipath (MP) with a reflection on point *x*.

Figure 5 shows a diagram where one of the many rays reflected on the surfaces in the environment also reaches the detector surface. Each time the light ray reflects on a surface, a fraction of the power is absorbed by the material, and the rest of the signal power is reflected. In this section, we propose to model the behavior of these reflections in the environment in order to compute the power signal that a detector will receive, based on adding other MP reflections to the LOS signal.

The reflections in the environment depend on multiple factors, the most important of which are:

- Angle of incidence.
- Light signal wavelength.
- Surface material features: roughness, color, form, etc.

**Figure 6:**
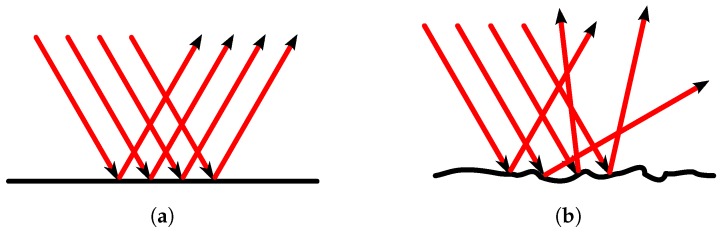
Light reflection on surface materials with different degrees of roughness. Flat surface (**a**) and rough surface (**b**).

Figure 6 presents two different cases of reflection considering surface materials with different degrees of roughness.

### 3.2. First Approximation

In our first approximation to the model, we assume the following hypothesis: the reflection on surface materials behaves as a unique temporary emitter oriented with the same angle, but opposite sign, with respect to the normal vector of the surface as the angle of incidence, and is on the same plane, as stated by Snell’s Law (same direction as a specular reflection).

Thus, the received power in a similar setting as the one shown in Figure 5, located at a distance $d_{\mathrm{RX}}$ from the point *x* and with an angle θ wrt the surface vector *x*, would be given by Equation (6):

(6) $$P_{\mathrm{RX}}=\frac{n+1}{2\pi }\frac{1}{d_{\mathrm{RX}}^{2}}\cos^{n}\left(\phi \right)A_{\mathrm{RX}}\cos\left(\alpha \right)P_{x}\beta$$

where $A_{\mathrm{RX}}$ is the effective receiver area and β is the reflected power ratio wrt the total received power $P_{x}$. As can be seen, the above Lambert Equation (5) has now been updated with the $\beta P_{x}$ emitted power. The *n* index and the β constant are different for each material. The *n* index represents how specular-diffuse the reflection of any surface material is.

Empirical tests showed that this particular model yields accurate results when considering rough or flat materials, but not with other kinds of surface. The reason behind this, analyzed in depth later, is that most materials have a reflection with more than one component; i.e., the real behavior will be given by the sum of multiple components, not only one Lambert equation.

### 3.3. Second Approximation

The second proposed hypothesis consists of a new complex model derived from the sum of multiple Lambert reflection components. The reflection on a material may be modeled as *N* independent emitters, each with its own orientation, power and *n* index. Now, the equation of the reflection becomes:

(7) $$P_{\mathrm{RX}}=\left[\sum_{i}^{N}a_{i}\left(\gamma \right)\frac{n_{i}\left(\gamma \right)+1}{2\pi }\cos^{n_{i}\left(\gamma \right)}\left(\theta_{i}\right)\right]\frac{1}{d_{\mathrm{RX}}^{2}}A_{\mathrm{RX}}\cos\left(\alpha \right)P_{x}\beta$$

where $a_{i}$ is the ratio between the power of *i* components with respect to the total power $P_{x}$. Therefore, the sum of all of the coefficients $a_{i}$ is equal to 1.0, as stated in Equation (8):

(8) $$\sum_{i}a_{i}=1$$

**Figure 7:**
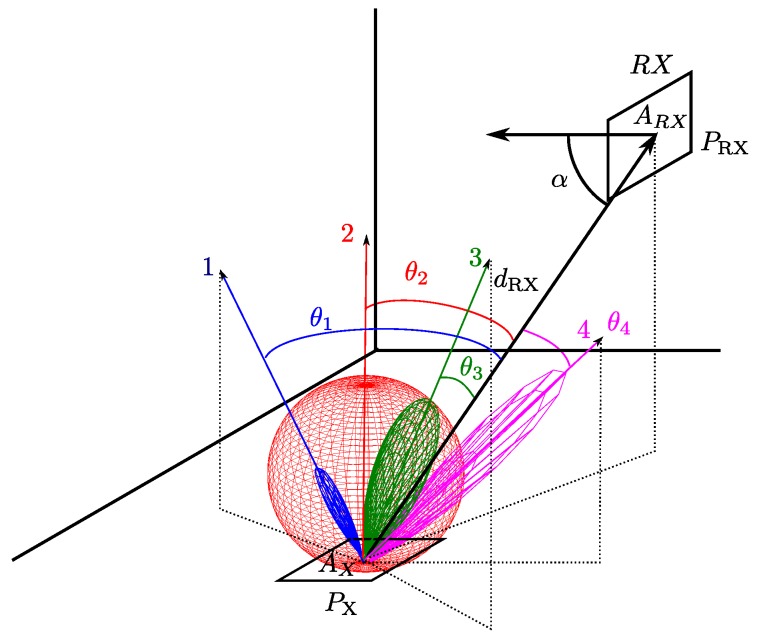
Example of $\theta_{i}$ angles of four different components.

Each of the components will be oriented at a certain angle $\theta_{i}$. These angles $\theta_{i}$ are obtained between the vector passing through the detector and point *x* (same origin for the *N* considered emitters) and the orientation vector for each of the *N* emitters. Figure 7 shows an example of the $\theta_{i}$ angles of four different components.

With this current model, each material is modeled with a given reflection β, *N* emitters, the $a_{i}$ values that represent the ratio between emitted power for each component *i* with respect to the total power $P_{x}$, its orientation and the emission diagram modeled as before, with the $n_{i}$ index.

After conducting empirical tests to study the validity of the proposal, we found that a model with more than three components did not incorporate additional information (for our application and others that do not require a high accuracy). Moreover, the difference between using two or three components was not significant (in the worst case, the maximum difference was 5%). Therefore, a final model of two components will be proposed and described in the next section.

### 3.4. Proposed Model: Two Components

After analyzing the first two hypotheses, in this section, we describe a reflection model composed of two components, one with a more diffuse behavior characterized by a low *n* index and another with a more specular behavior with a high *n* index.

**Figure 8:**
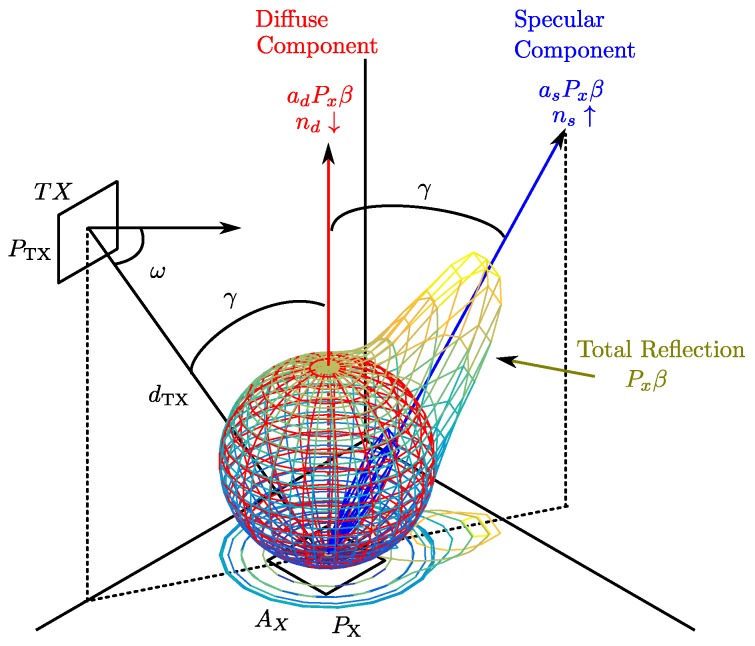
Reflection model.

Based on the power $P_{x}$ received in a given area $A_{x}$ (centered on a point *x*), the model considers that the power reflected on the surface centered on point *x*, whose value will be $P_{x}\beta$, will have an emission pattern equal to that of two independent emitters, one with a low *n* index and the other with a high *n* index, located at the same point *x*, but with a different orientation. The diffuse component will be oriented with respect to the normal reflection surface and will emit a power $a_{d}P_{x}\beta$ with an emission diagram characterized by the value of $n_{d}$ (very close to what would be a pure diffuse reflection), while the specular component will be oriented at an angle γ opposite to the incident ray with respect to the normal (surface vector), with a power $a_{s}P_{x}\beta$ and an emission diagram characterized by $n_{s}$. Figure 8 shows an example of the reflection at a given point *x*. The diffuse component is shown as a sphere; the specular component is shown in blue; and the total reflection is graded from cyan to yellow.

For the sake of simplicity, throughout this paper, the term “diffuse component” will be used to refer to the component with the most similar behavior to a diffuse one. Similarly, the reflective component with the highest *n* index will be called the specular component.

Therefore, according to the diagram shown in Figure 5, the reflected power on a surface material, P(γ,ϕ,θ), with a given angle of incidence γ wrt to the surface normal vector, reaching the detector at a given angle θ wrt the normal and ϕ angle wrt the maximum reflection ray (opposite to the incident ray), at a distance $d_{\mathrm{RX}}$ and with an effective detector area $A_{\mathrm{RX}}$, will be the sum of the two components in the current model:

(9) $$P\left(\gamma ,\phi ,\theta \right)=P_{d}\left(\gamma ,\theta \right)+P_{s}\left(\gamma ,\phi \right)$$

where diffuse and specular power values are given by the Equations (10) and (11), respectively:

(10) $$P_{d}\left(\gamma ,\phi ,\theta \right)=\left(\frac{n_{d}\left(\gamma \right)+1}{2\pi }\right)a_{d}\left(\gamma \right)P_{x}\beta \cos^{n_{d}\left(\gamma \right)}\left(\theta \right)A_{\mathrm{eff}}\frac{1}{d_{\mathrm{RX}}^{2}}$$

(11) $$P_{s}\left(\gamma ,\phi ,\theta \right)=\left(\frac{n_{s}\left(\gamma \right)+1}{2\pi }\right)a_{s}\left(\gamma \right)P_{x}\beta \cos^{n_{s}\left(\gamma \right)}\left(\phi \right)A_{\mathrm{eff}}\frac{1}{d_{\mathrm{RX}}^{2}}$$

Therefore, the complete expression for the power is given in Equation (12):

(12) $$P\left(\gamma ,\phi ,\theta \right)=\left[a_{d}\left(\gamma \right)\left(\frac{n_{d}\left(\gamma \right)+1}{2\pi }\right)\cos^{n_{d}\left(\gamma \right)}\left(\theta \right)\;+a_{s}\left(\gamma \right)\left(\frac{n_{s}\left(\gamma \right)+1}{2\pi }\right)\cos^{n_{s}\left(\gamma \right)}\left(\phi \right)\right]A_{\mathrm{eff}}\frac{1}{d_{\mathrm{RX}}^{2}}P_{x}\beta$$

where $A_{\mathrm{eff}}$ is the effective receiver area that can be expressed as $A_{\mathrm{eff}}=A_{\mathrm{RX}}\cos\left(\alpha \right)$, where $A_{\mathrm{RX}}$ is the receiver area and α the angle between the incident ray on the detector wrt the surface normal vector.

Given that the sum of power emitted by the specular and diffuse components must be equal to the reflected power on the surface material, the expression in Equation (13) must be fulfilled:

(13) $$a_{d}\left(\gamma \right)+a_{s}\left(\gamma \right)=1$$

due to the fact that both represent the total reflected power for each of the two considered components.

**Figure 9:**
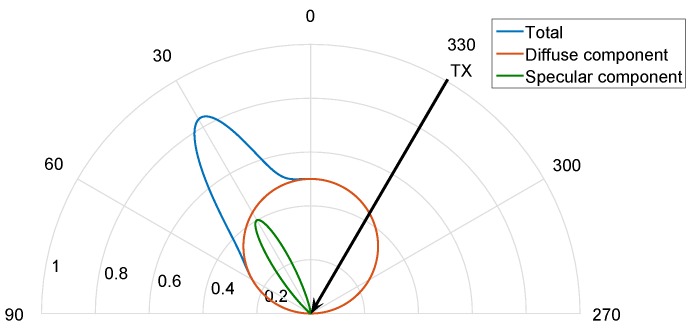
Example of a reflection pattern emission based on two components.

To facilitate understanding of the following descriptions, a reflection example with these two components is shown in Figure 9. The emitter is located at an angle $\gamma =330^{\circ }$. The diffuse component (in red) is oriented along the surface normal vector, in this case $0^{\circ }$, with a wide emission diagram characterized by a low *n* index. The specular component (in green) is oriented along the $30^{\circ }$ angle (symmetric to the normal vector) and has a narrower emission diagram characterized with a high *n* index. The sum of both reflection components is shown in blue.

The parameters from Equation (12) that remain constant for different surface materials are the effective sensor area $A_{\mathrm{eff}}$, the distance between the reflection point and the detector $d_{\mathrm{RX}}$ and the power $P_{x}$ of the incident ray on the reflection point *x*. The parameters that change for different surface materials are the diffuse and specular components ($a_{d}$ and $a_{s}$ respectively), their emission *n* indexes ($n_{d}$ and $n_{s}$) and the specific reflection of the material (β).

There are important differences between the proposed model and the Phong model; firstly, the Phong model considers that the diffuse component has an index $n_{d}=1$, whereas in our proposition, this value will be different for each surface material. However, the main difference is that in our case, the parameters $a_{d}$, $a_{s}$, $n_{d}$ and $n_{s}$ are variable depending on the angle of incidence, a behavior that has been seen in real reflections. Therefore, this latter model presents a better fit with the real behavior of light ray reflections in the environment. On the other hand, a new function must be characterized to adjust these parameters along the different angles of incidence.

One simple approximation for such a function is given in Equation (14):

(14) $$z=u_{z}\cos^{v_{z}}\left(\gamma \right)\;$$

where *z* is the name of each variable parameter and $u_{z}$ and $v_{z}$ are the coefficients to be fitted.

Hence, the functions for parameters $a_{d}$, $a_{s}$, $n_{d}$ and $n_{s}$ are the following:

(15) $$a_{s}=u_{\mathrm{as}}\cos^{v_{\mathrm{as}}}\left(\gamma \right)\;$$

(16) $$a_{d}=1-a_{s}=1-u_{\mathrm{as}}\cos^{v_{\mathrm{as}}}\left(\gamma \right)\;$$

(17) $$n_{d}=u_{\mathrm{nd}}\cos^{v_{\mathrm{nd}}}\left(\gamma \right)\;$$

(18) $$n_{s}=u_{\mathrm{ns}}\cos^{v_{\mathrm{ns}}}\left(\gamma \right)\;$$

Therefore, the expression for the reflection model as a function of the angle of incidence γ is shown in Equation (19):

(19) $$P\left(\gamma ,\phi ,\theta \right)=\left[p_{d}\left(\gamma ,\theta \right)+p_{s}\left(\gamma ,\phi \right)\right]K$$

where the subterms $p_{d}$ and $p_{s}$ are given by Equations (20) and (21), respectively:

(20) $$p_{d}\left(\gamma ,\theta \right)=\left(1-u_{\mathrm{as}}\cos^{v_{\mathrm{as}}}\left(\gamma \right)\right)\left(\frac{u_{\mathrm{nd}}\cos^{v_{\mathrm{nd}}}\left(\gamma \right)+1}{2\pi }\right)\cos^{u_{\mathrm{nd}}\cos^{v_{\mathrm{nd}}}\left(\gamma \right)}\left(\theta \right)$$

(21) $$p_{s}\left(\gamma ,\phi \right)=u_{\mathrm{as}}\cos^{v_{\mathrm{as}}}\left(\gamma \right)\left(\frac{u_{\mathrm{ns}}\cos^{v_{\mathrm{ns}}}\left(\gamma \right)+1}{2\pi }\right)\cos^{u_{\mathrm{ns}}\cos^{v_{\mathrm{ns}}}\left(\gamma \right)}\left(\phi \right)$$

and *K* represents the grouped factor parameters that are constant:

(22) $$K=A_{\mathrm{eff}}\frac{1}{d_{\mathrm{RX}}^{2}}P_{x}\beta$$

In Equation (19), the subterm *K* could be reduced to the β parameter if particular values for the effective area, $P_{x}$, and $d_{\mathrm{RX}}$ are considered. In our case, we chose not to reduce the number of parameters and to use the parameter *K*, since we could obtain the rest of the parameters for any value of the effective area, $P_{x}$, or $d_{\mathrm{RX}}$.

When using the reflection model, the subterm *K* will be extracted and used to obtain the specific material reflection value, β, since the emitted power $P_{\mathrm{TX}}$ and as a consequence the power $P_{x}$ are known, and the rest of the parameters can be measured with sufficient accuracy.

**Figure 10:**
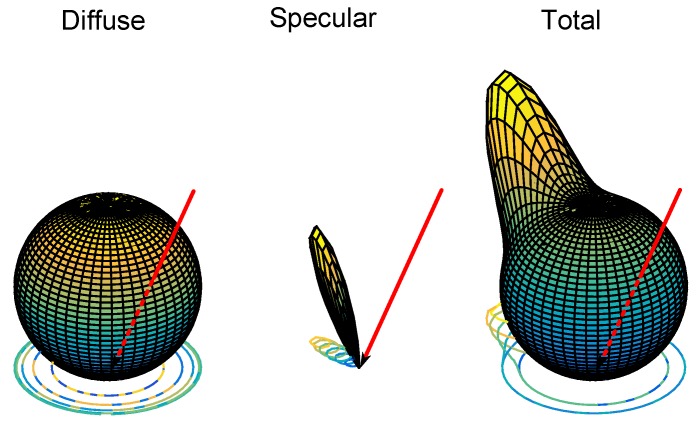
Example emission pattern: 3D diagrams for a two-component model.

The proposed reflection model considers that the reflection diagram of a particular surface material is the sum of the 3D diagrams of the diffuse and specular components. The 3D diagrams for each component are a revolution volume along their orientation axes. Figure 10 shows the 3D reflection diagrams for the same example considered in Figure 9.

Thus, in the final expression, it can be seen that there are seven different and independent coefficients: $u_{as}$, $v_{as}$, $u_{nd}$, $v_{nd}$, $u_{ns}$, $v_{ns}$ and *k*; which are necessary to obtain from experimental measurements and an optimization or adjustment method.

#### Limitations of the Proposed Reflection Model

**Figure 11:**
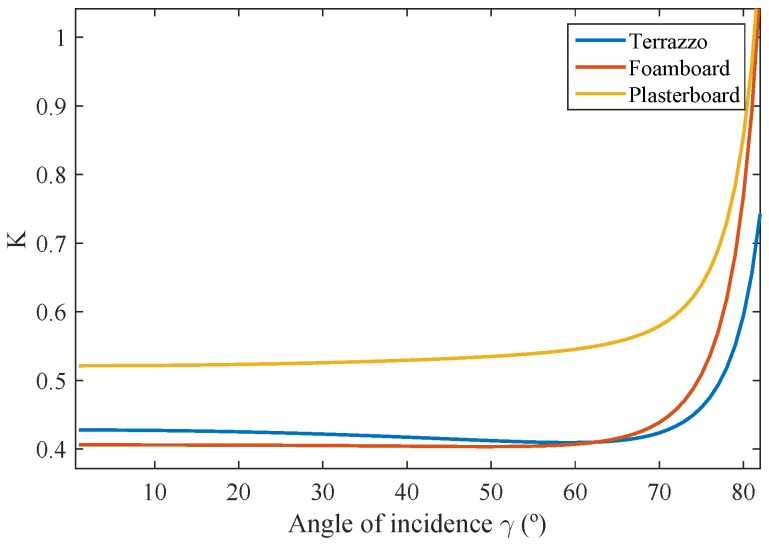
Parameter *K* as a function of the angle of incidence.

After performing the first experimental measurements, we observed that the reflection parameter β was not constant, but changed with the angle of incidence. However, for angles of incidence of less than 70 degrees, it can be approximated with great accuracy to a constant. To demonstrate this, Figure 11 shows the *K* parameter as a function of the angle of incidence for the three different materials that will be used throughout this paper: terrazzo, a foam board and a plasterboard. Experimental measurements were also performed with other materials, but these three materials are included as being representative of different types of reflection. To obtain the value of the parameter *K* as a function of the angle of incidence, the fitting functions for the parameters $a_{d}$, $a_{s}$, $n_{d}$, $n_{s}$ and *K* were adjusted from the experimental measurements of $P\left(\gamma ,\phi ,\theta \right)$ along angle θ, computed separately for each angle of incidence.

Because the value of *K* is equal to $A_{\mathrm{eff}}\frac{1}{d_{\mathrm{RX}}^{2}}P_{x}\beta$, the variation in the *K* parameter with the angle of incidence is due solely to the reflection factor β, since this is the only parameter that can vary with the angle of incidence.

## 4. Methodology for Obtaining Parameter Values

In order to find the value of the different coefficients that form the reflection model, it is necessary to carry out a series of experimental measurements, for which suitable measurement instruments are required. In this section, we first show a simple instrument for taking empirical measurements, then we analyze the data obtained and propose a method to obtain the values of the model parameters from them.

### 4.1. Data Acquisition Tool and Procedure

The main features of the measuring tool and a description of the instruments used are given below.

The characteristics that the tool must have to perform the required experimental measurements are:

- A 180 degree movement of the emitter and detector on the same plane. The emitter and the detector must always be pointing toward the same point on the surface material analyzed, regardless of the angle.
- The emitter must have an emission spot narrow enough to be capable of assuming that the power received on the material can then be considered a reflection point emitter.

**Figure 12:**
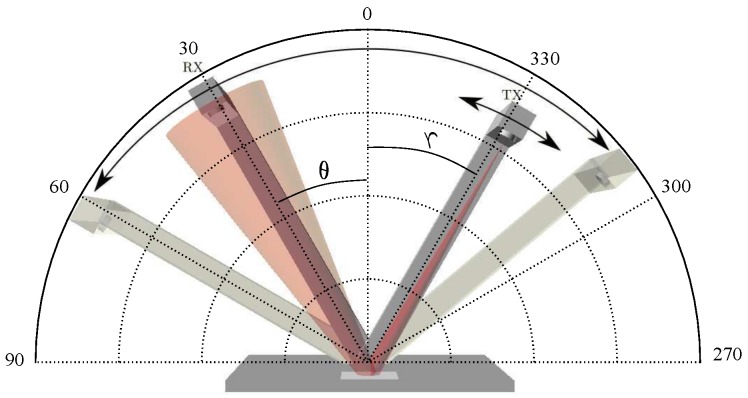
A schematic diagram of the tool used.

A schematic diagram of the tool used is shown in Figure 12.

The material sample to be analyzed is placed on the plane containing the axis of rotation of the emitter and the detector. This is achieved by having the same reference system for all angles.

**Figure 13:**
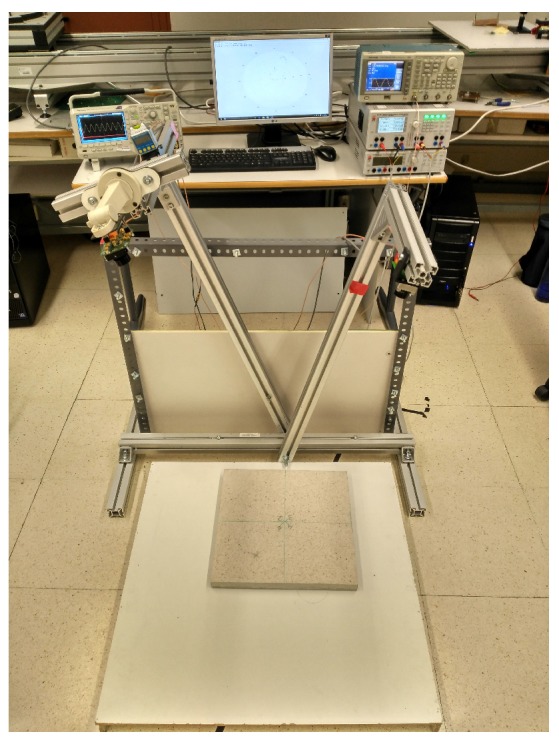
Measurement tool used during the project.

In our case, the tool was made of aluminum (Figure 13) to endow it with the necessary rigidity and stability. Both the emitter and the detector were placed at a distance of about one meter away from the material to be analyzed to obtain good accuracy in the measurement of the angles.

The procedure for obtaining the experimental measurements is described below. An optical sinusoidal signal is emitted at 940 nm at a frequency of 8 MHz, and the detector receives the NIR signal reflected by the material to be analyzed. The output voltage of the detector is captured with an oscilloscope, which internally performs an FFT of the signal and returns the rms value of the 8-MHz component to an attached PC. To reduce any biases in the measurement and noise from different sources, which is considered Gaussian white noise with zero mean, for each detector position, the signal is acquired for a long period of time, so that the rms value is averaged.

The sensor generates a current from the optical power received, which depends on the value of the detector responsivity in Amper per Watt (A/W). Next, this current is transformed into voltage using a series of linear amplifiers; the rms voltage obtained can be considered proportional to the received power. Therefore, although we will discuss power in the following sections, what is really obtained from the detector is an rms voltage value.

The optical power value received by the detector will be calculated for a set of angles of incidence with the above measuring tool.

The angle of incidence $\left(\gamma \right)$ ranges from −10 to −60 degrees in steps of 10 degrees. For each angle of incidence, a sweep of measurements is performed along θ. The value of θ is modified from −80 to 80 degrees with a non-equidistant separation, taking more measurements for the more relevant angles, which according to the proposed model are situated at angles close to −γ and taking fewer values for the rest. Because the *K* parameter for a ∣θ∣ above 70 degrees is no longer constant, the model will not fit perfectly for measurements performed above this value.

### 4.2. Data Obtained from Experimental Measurements

**Figure 14:**
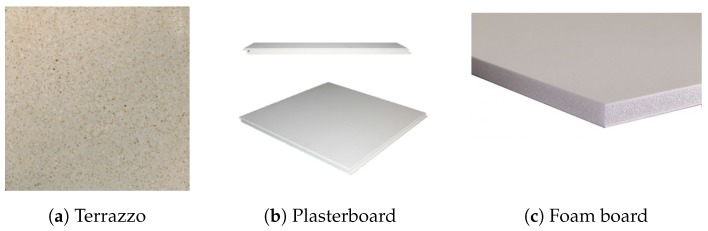
Materials analyzed in this study.

Three materials of very different composition were analyzed: typical terrazzo, foam board and plasterboard used to cover ceilings. Figure 14 shows the three materials used.

**Figure 15:**
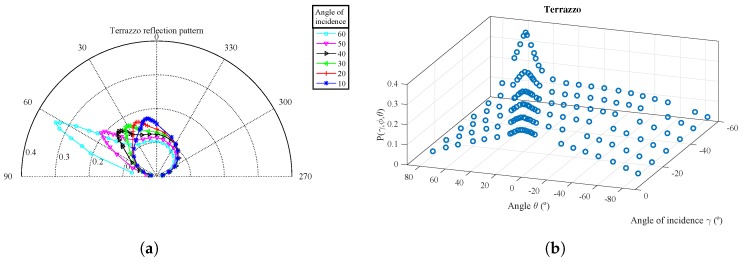
Experimental measurements for $P\left(\gamma ,\phi ,\theta \right)$ as a function of θ for different values of γ for the terrazzo. (**a**) Polar coordinates; (**b**) 3D diagram.

**Figure 16:**
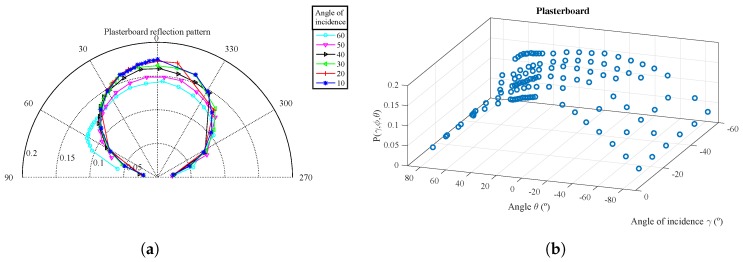
Experimental measurements for $P\left(\gamma ,\phi ,\theta \right)$ as a function of θ for different values of γ for the plasterboard. (**a**) Polar coordinates; (**b**) 3D diagram.

**Figure 17:**
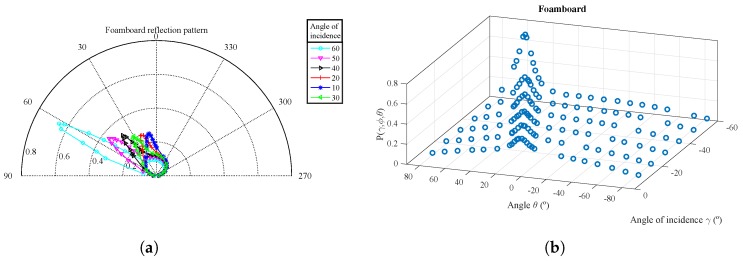
Experimental measurements for $P\left(\gamma ,\phi ,\theta \right)$ as a function of θ for different values of γ for the foam board. (**a**) Polar coordinates; (**b**) 3D diagram.

For each surface material, Figure 15, Figure 16 and Figure 17 show the parameter vales obtained from the experimental measurements in polar coordinates and in 3D. The parameter values $P\left(\gamma ,\phi ,\theta \right)$ are shown as a function of θ for different values of the angle of incidence γ.

The polar representation shows the value of $P\left(\gamma ,\phi ,\theta \right)$ for each of the angles of incidence measured with a different color curve and marker. Each marker shows the exact point where the measure was taken.

The 3D diagram shows $P\left(\gamma ,\phi ,\theta \right)$ as a function of both θ and γ angles. As the measurements were captured with the angles of incidence and reflection on the same plane, the ϕ angle can be extracted from the following relationship: ϕ=−γ−θ.

From the measurements obtained for the terrazzo and foam board, it can be seen that the reflection diagram is composed of at least of two components. In Figure 15a and Figure 17a, the polar coordinates for each angle of incidence clearly show that there is one component with a diagram similar to a Lambertian diagram (with an index value n=1) and another component with a narrower diagram, oriented along an angle equal to γ that is added to the former one.

Figure 9 depicts a similar behavior, directly confirming the hypothesis for our proposed modelization. It can also be seen that the distribution of power between the diffuse and specular components varies with the angle of incidence. As this latter increases, the power of the specular component increases while the diffuse emission diagram becomes wider and less important.

In the case of the reflection diagram for the plasterboard, it can be seen that unlike the previous materials, this presents practically a single component with a similar behavior to a Lambertian one, because the specular component is almost null. In this case, our extracted model coefficients give greater weight to the more diffuse component.

### 4.3. Method for Obtaining the Coefficient Values

The values for the seven model parameters are obtained by data fitting in the Equation (19) using experimental measurements.

The error between the adjusted model and actual reflection behavior will depend on both the number of measurements chosen and the angle where these measurements are obtained. Therefore, it is necessary to establish a method that makes it possible to obtain the value of the parameters with the minimum possible number of measurements while obtaining an acceptable error between the model and the real system.

We used genetic algorithms to determine how many measurements to take at which angles of incidence and reception, analyzing all available measurements to minimize both the number of points used in the function fitting and the error between the model adjusted with these points for all available experimental measurements. Therefore, the variables to be minimized will be the number of measures *N* and the value of $1-R^{2}$, where $R^{2}$ is the coefficient of determination R-square between the curve adjusted with these *N* points and all available experimental measurements.

**Figure 18:**
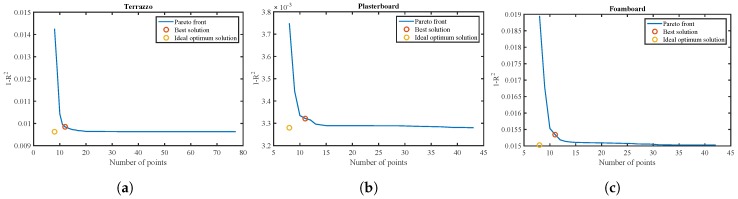
Pareto frontier for the three analyzed materials. (**a**) Terrazzo; (**b**) plasterboard; and (**c**) foam board.

Figure 18 shows the Pareto frontier for the terrazzo, the plasterboard and the foam board.

The *x* axis has the number of points used in the function fitting. The *y* axis represents the value $1-R^{2}$. An orange marker shows the ideal point for the best fit and minimum number of measurements used. This point represents a non-feasible solution that does not belong to the fitted curve; therefore, a marker is shown in red at the closest point that will be the best feasible solution.

The optimum number of points is 11 points for the plasterboard and foam board and 12 points for the terrazzo. In order to generalize the number of points for any kind of material present in the environment, a number of 12 points is chosen for the data fitting. Note that it is more important to choose these points correctly than the number of points. Therefore, once the number of points introduced in the adjustment has been defined as 12, the next step is to determine the optimal ones.

**Figure 19:**
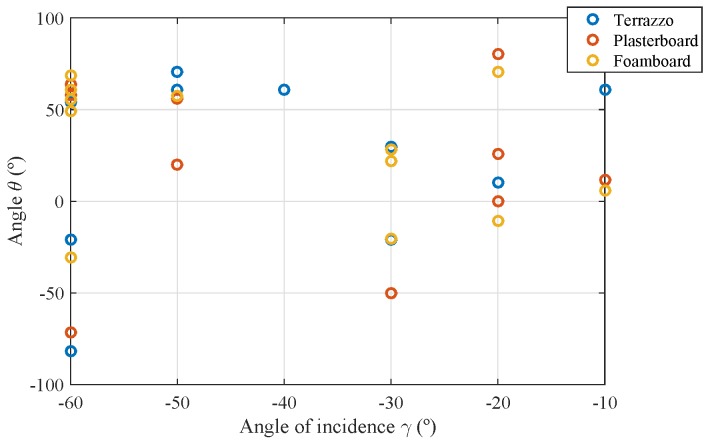
Location for the optimal 12 points considering the three types of surface materials.

Figure 19 shows the location of the 12 optimal points considering the three surface materials. The *x* axis represents the angle of incidence γ, and the *y* axis shows the value for the reception angle θ. The optimal solution given by optimization via genetic algorithms locates the angles in those for which measurements are available.

As can be seen in Figure 19, the 12 optimal points are located in different places for each of the three types of surface material. Even so, we can see how the optimal points are distributed for the different angles of incidence, and for each of these, more points are considered in angles θ located in values where the reflection is maximum, that is close to −γ.

Starting from the solutions obtained using genetic algorithms, we propose a methodology to obtain the values of the model parameters, which is valid for most kinds of material with only 12 measurements recovered from certain angles. The method is as follows:

- Select three different distant angles of incidence.
  (23) $$\gamma_{1},\;\gamma_{2}\;\mathrm{and}\;\gamma_{3}\;\mathrm{such}\;\mathrm{that}\;\gamma_{1}<\gamma_{2}<\gamma_{3}$$
- Obtain the measurement of the received power at five different points for the extreme angles (min $\gamma_{1}$ and max $\gamma_{3}$).
  (24) $$\theta_{m1},\;\theta_{m2},\;\theta_{m3},\;\theta_{m4}\;\mathrm{and}\;\theta_{m5}$$
  (25) $$\theta_{M1},\;\theta_{M2},\;\theta_{M3},\;\theta_{M4}\;\mathrm{and}\;\theta_{M5}$$
- Obtain two measurements for the central value of the angles of incidence.
  (26) $$\theta_{c1}\;\mathrm{and}\;\theta_{c2}$$

The received power measurements are performed using the following criteria:

- For the maximum and minimum test angles of incidence, up to five different θ angles must be selected.
  − One angle must be equal to the opposite value of the angle of incidence −γ, so as to detect the peak value of the specular component:
    (27) $$\theta_{m1}=-\gamma_{1}$$
    (28) $$\theta_{M1}=-\gamma_{3}$$
  − Two other angles must be separated from the first one by ±10 degrees, so as to detect/model the width of the specular component.
    (29) $$\theta_{m2},\theta_{m3}=\theta_{m1}\pm 10^{\circ }$$
    (30) $$\theta_{M2},\theta_{M3}=\theta_{M1}\pm 10^{\circ }$$
  − The last two θ angles must be chosen, so as to be distant from the first one, for example a difference of 60 degrees. In the case that one of these values exceeds the maximum value that the tool can set for the θ angle, the extreme angle value must be selected. With these distant angles, the fitting will better adjust the diffuse component.
    (31) $$\theta_{m4},\theta_{m5}=\theta_{m1}\pm 60^{\circ }$$
    (32) $$\theta_{M4},\theta_{M5}=\theta_{M1}\pm 60^{\circ }$$
- For the central angle of incidence, two different θ angles must be set distant from the orientation of the specular component (for example, 20 degrees).
  (33) $$\theta_{c1}=\gamma_{2}+20^{\circ }$$
  (34) $$\theta_{c2}=-\gamma_{2}+20^{\circ }$$

Note that with the proposed methodology, we obtained slightly worse accuracy than with the optimal points selection method using genetic algorithms, but the methodology yields an accurate reflection model for different surface materials using only 12 measurement points and always employing the same procedure.

## 5. Results and Adjustment of the Proposed Method

In this section, we will describe how the proposed reflection model is adjusted to the experimental measurements. For this, the value of the model coefficients is obtained following the 12-point procedure described earlier. Then, we will compare these results with those obtained when using a higher number of points to determine the model parameter values. Finally, we will compare our proposed reflection model with other state-of-the-art models.

### 5.1. Results of the Proposed Method

Below, we present the adjustment results for the reflection model coefficients obtained using the 12-point procedure.

We used a total of 132 experimental measurements for the terrazzo and foam board and 102 measurements for the plasterboard. Six angles of incidence were selected between −10 and −60 degrees. For each angle of incidence, we chose a total of 22 or 17 angles θ between −70 and 70 degrees. Because plasterboard is a more uniform material, fewer experimental measurements were chosen.

**Figure 20:**
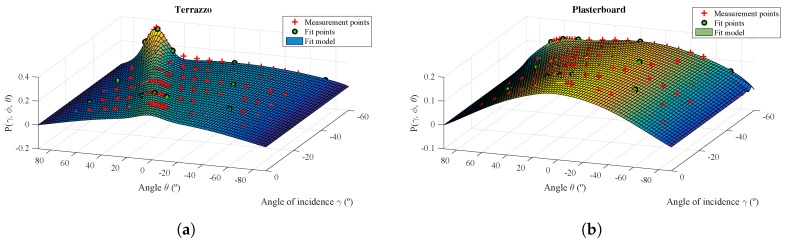
3D diagram for the model parameters $P\left(\gamma ,\phi ,\theta \right)$ as a function of the γ and θ angles. (**a**) Terrazzo; (**b**) plasterboard.

Table 1 shows the different γ and θ angles selected to obtain the measurements:

In this section, we present the results for the terrazzo and the plasterboard, as examples of one very specular material with two clearly distinct components and another material with a highly diffuse reflection behavior with one of the components (specular) almost canceled out. Figure 20 shows the reflection model adjusted as a function of the angle θ and γ. The adjustment procedure used the 12 power measurements obtained in the angles shown in Table 1. These measurements are shown in the figure as green circles. The data points shown as red crosses represent all of the data obtained from the experimental measurements; these were not used to calculate the model coefficients, but are shown here to compare the reflection model with the measured real values.

**Figure 21:**
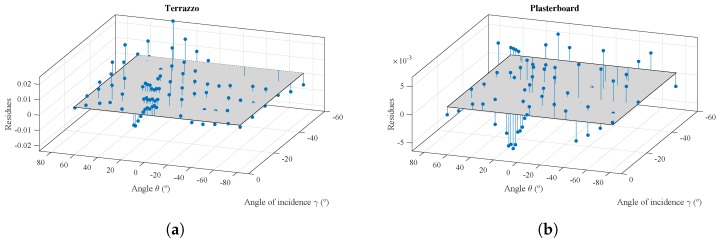
3D diagram with errors between the values adjusted using the reflection model and the experimental measurements. (**a**) Terrazzo; (**b**) plasterboard.

As can be seen, the reflection model fits the values of the experimental measurements on two surface materials, terrazzo and plasterboard, that present very different behavior. To observe this adjustment in more detail, Figure 21 shows the errors between the adjusted reflection model and the values of the experimental measurements shown in Figure 20 as red markers.

**Figure 22:**
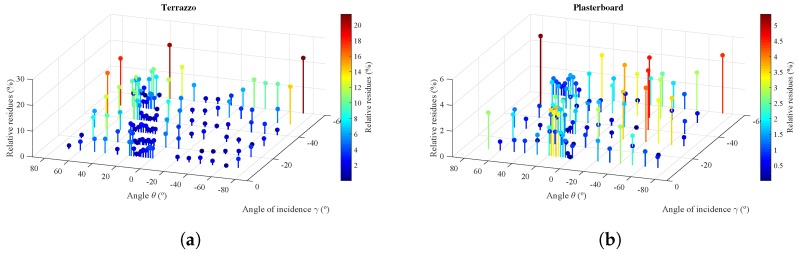
3D diagram of the normalized residues between model and experimental measurements. (**a**) Terrazzo; (**b**) plasterboard.

To provide a reference for the relative value of the residues, these are shown in Figure 22. Relative residues were obtained from the difference between the reflection model and the experimental measurements divided by the value of the experimental measurement.

**Figure 23:**
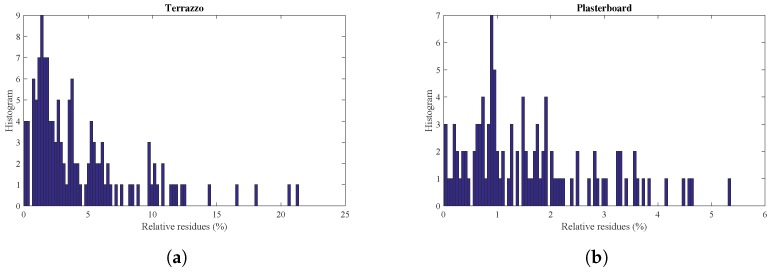
Histogram of normalized residues. (**a**) Terrazzo; (**b**) plasterboard.

To illustrate the range of errors between the reflection model and the measured values, Figure 23 shows a histogram of the residues. In the case of the terrazzo, most of the residues are located below 8%, with some larger outliers that might be errors in the experimental data measurements. In the case of the plasterboard, the residues are lower, all below 5%.

Table 2 shows the adjustment value R-square, the mean quadratic error (MSE) and its root-square value (RMSE).

To sum up, Table 3 shows the parameters values of the reflection model for the materials analyzed throughout the paper.

As noted before, the reflection model parameter values are obtained from 12 experimental measurements, each consisting of an angle triplet θ, γ and ϕ and a measured power value corresponding to this triple angle configuration. By applying optimization algorithms, we can obtain the values of the other seven parameters that best fit the model equation (Equation (19)) to these experimental measurements.

The algorithm to obtain the model parameters has been executed in MATLAB R2015 on a desktop PC with an Intel Core 2 Duo E8400 with 8.00 GB DDR2 RAM. Once the 12 measurements are obtained (according to the proposed method), the algorithm to obtain the parameter values was repeated 10,000 times and then averaged. This methodology has been repeated for each material under analysis. The obtained results are shown in Table 4.

As shown before, the execution time depends on the material under analysis. This is because we are using iterative algorithms to obtain the model fitting, which depends on the supplied initial information for each material and the convergence to the final solution.

### 5.2. Comparison with the Results Obtained Using a Different Number of Points to Determine Model Parameter Values

In this section, we will describe the adjustment between the reflection model and the experimental measurements, obtaining the parameter values using three different methods. The methods employ a different number of data points in the adjustment:

- Method A: Use every available measurement. In our experimental tests, we have a complete set of 132 data in terrazzo and foam board and a set of 102 data in plasterboard.
- Method B: From all the experimental measurements, use a genetic algorithm to select the points that minimize both the error and the number of items of data used in the adjustment. Therefore, from the 132 or 102 measurement points, we used the 12 data points shown in Figure 19 to fit the model.
- Method 12: This is our proposed method, which uses the data obtained from the 12-point procedure. The measurements are taken at the angles shown in Table 1.

Table 5 shows the value of R-Square for the three above methods together with the considered number of points used to obtain the value of the reflection model parameters.

Table 5 shows that the proposed 12-point method obtains a data fit that is very close to the best method, namely the genetic algorithm procedure to select the 12 optimal points out of 132 available items of data.

### 5.3. Comparison with Other State-Of-The-Art Models

In this section, we will compare our proposed reflection model with other existing reflection models.

Firstly, we will compare our proposed reflection model with the Phong model. The Phong model was chosen because like ours, it is a mixed reflection model, using two components, and because its parameters can also be obtained from the given experimental data measurements.

The adjustment results will be analyzed for the same three surface materials that have been discussed throughout the article, terrazzo, plasterboard and foam board. To perform the comparison, a separate error parameter will be obtained for each angle of incidence. The error parameter is a relative error that considers the area between the function of the calculated model and the experimental measurements in polar coordinates, divided by the area of the experimental measurements.

**Figure 24:**
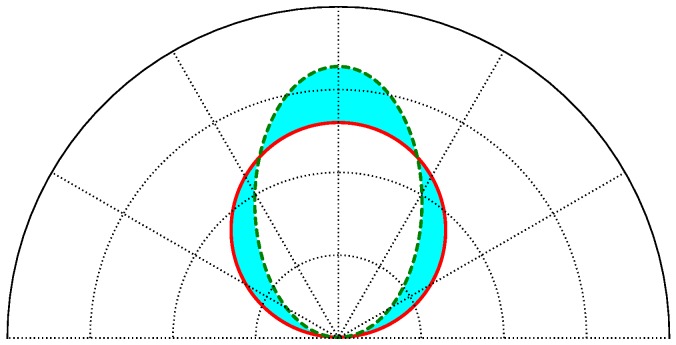
Example of error calculation.

Figure 24 gives an example to illustrate how the error is calculated. The continuous red line represents the experimental measurements, while the discontinuous green line represents the function obtained with the reflection model. The error is calculated as the blue area divided by the area of the red circle.

Since the Phong model does not vary the behavior of the diffuse and specular components as a function of the angle of incidence, its parameters will be adjusted for one test angle of incidence, for example $\gamma =10^{\circ }$.

**Figure 25:**
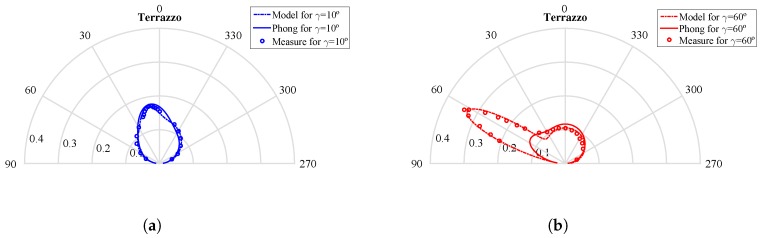
Polar coordinate system with ground-truth experimental measurements (circle markers), curve fitting from our proposed model (dash-dotted line) and curve fitting for the Phong model (solid line). (**a**) Angle of incidence $\gamma =10^{\circ }$; (**b**) angle of incidence $\gamma =60^{\circ }$.

Using a polar coordinate system, Figure 25 shows the ground-truth experimental measurements (with circle markers), the curve fitting given by our proposed model (dash-dotted line) and the curve fitting for the Phong model (solid line)), as a function of θ for two different angles of incidence $\gamma =10^{\circ }$ and $\gamma =60^{\circ }$, considering the terrazzo surface material.

As can be seen, the Phong model is not capable of adjusting the variation in the emission diagram of the diffuse and specular components as a function of the angle of incidence. For the angle of incidence used to obtain the parameters for the Phong model, $\gamma =10^{\circ }$, the adjustment with the experimental measurements is satisfactory. However, as the angle of incidence increases, the diagram obtained with the Phong model does not conform to reality. In contrast, our proposed reflection model is capable of modeling the variation in these reflection diagrams with two components as a function of the angle of incidence, obtaining a good fit for the different angles.

Table 6 shows the errors for the three materials considering six different angles of incidence for the proposed model and the Phong model.

It can be observed that the error becomes greater as the angle of incidence increases because the Phong model does not consider the variation in the diffuse and specular components as a function of the angle of incidence. In contrast, the errors obtained using our proposed reflection model are low and are not dependent on the angle of incidence.

The review in [17] analyzes the most important reflection models, conducting a comparison of the following models: He–Torrance, Beckmann–Spizzichino, Torrance–Sparrow, Nayar and Schlick with respect to the ground-truth data obtained from a gonioreflectometer for three different materials.

The surface materials used in this analysis were: chromium (specular material), veneer (material with two reflection components, diffuse and specular ones) and a painted surface (more diffuse behavior).

The results of model accuracy with respect to the data measured with the gonioreflectometer are presented as a relative error, calculated in a similar manner as discussed above. Table 7 shows the comparison obtained in [17]. The values between parentheses are error values obtained by introducing modifications in the analyzed models.

Since the material samples with which the tests described in [17] were conducted are not currently available, it is not possible to perform a direct comparison between our model and these model results.

However, as the error calculation is the same in [17] as in Table 6, it is possible to draw some conclusions. These models present large error values with all three materials considered, yielding 40% errors for chromium, between 5% and 22% for veneer and 15% for the painted surface.

The painted material might be considered comparable to our plasterboard surface, for which our model obtained a maximum error below 3%. In the other materials presented in our paper, the errors were always below 6%.

## 6. Conclusions

In this paper, we have presented a new model of the behavior of IR light reflections in indoor environments for different surface materials. We have also presented a simple methodology for adjusting the model parameters.

Two different models have been described; an initial one that does not present a complete fit for all types of material, and a second, much more complete model that fits any type of surface material. This second model uses N components to characterize the reflection on different materials; real tests indicated that the accuracy obtained with only three components is sufficiently high for most applications. Reducing the number of components to two greatly simplifies the model without significantly affecting measurement accuracy, which remains sufficient for LPS applications. The best feature of the two-component model is that it greatly simplifies the computation of parameters.

Thus, we propose a two-component model (one component presenting specular and the other diffuse behavior). The index parameters of both components’ $n_{s}$ and $n_{d}$ variables must be fitted with the rest of parameters. This considers that these reflection indexes and the two components’ reflection powers $a_{d}$ and $a_{s}$ vary with the angle of incidence. We found that the diffuse component widens and loses power with larger angles of incidence. The specular component has the opposite effect.

The proposed fitting function for other variable parameters is the same for all of them, and we have shown that their coefficients can be perfectly adjusted following that expression.

The methodology for adjusting the coefficient values is based on genetic algorithms. We performed experimental tests on different materials and analyzed the minimum number of experimental measures that is possible to use so as to obtain an optimal result for the calculation of parameters and coefficients. In addition, we determined the best angles of incidence and reflection to use for measuring each surface material in an indoor environment. As a result, we defined a procedure to obtain 12 measurements and then used them to adjust all parameters and coefficients in the proposed model.

We compared the proposed model with real data obtained from 144 different angle combinations. As reported in the Results section, the errors do not depend on the type of material or on angles of incidence and reflection; accuracy is sufficiently high for indoor LPS; and the curve obtained with the model for different materials fits the real measured values perfectly, which has also been verified through residues’ calculation.

From the comparison with other existing state-of-the-art models, we conclude that our proposed model yields better accuracy, is suitable for any kind of surface material and, above all, does not depend on the angles of incidence and reflection.

The proposed model can be applied to characterize the multipath behavior of optical signals in applications such as indoor positioning and VLC communications. In fact, there is a current work in progress based on this reflection model, which computes off-line the estimated behavior of the multipath rays considering that the room geometry and objects are known so as to try to later mitigate the multipath effects in real time and improve the positioning obtained by a PSD-IRED-based system. Additionally, a simulation tool to perform these calculations automatically from a known room geometry is being developed.

## Figures and Tables

**Table 1:** Values of θ and γ angles selected to obtain the measurements in 12 points.

Angle of Incidence γ (°)	Angle θ (°)				
−20	20	10	30	−40	70
−40	−20	60			
−60	60	50	70	0	−70

**Table 2:** R-square, RMSE and MSE of the proposed model for different materials.

Material	R-Square	RMSE	MSE
Terrazzo	0.9831	0.0074	$5.4346\times 10^{-5}$
Plasterboard	0.9928	0.0026	$7.0095\times 10^{-6}$
Foam board	0.9742	0.0188	$3.5177\times 10^{-4}$

**Table 3:** Model parameter $P\left(\gamma ,\phi ,\theta \right)$ values for the different materials.

Material	$u_{\mathrm{as}}$	$v_{\mathrm{as}}$	$u_{\mathrm{nd}}$	$v_{\mathrm{nd}}$	$u_{\mathrm{ns}}$	$v_{\mathrm{ns}}$	K
Terrazzo	0.01713	−2.379	1.021	0.4283	34.55	−0.3772	0.4142
Plasterboard	0.001284	−4.911	1.083	0.4636	33.41	1.382	0.5215
Foam board	0.01742	−3.103	1.457	1.005	102	0.731	0.3879

**Table 4:** Execution time of the optimization algorithms for different materials.

	Terrazzo	Plasterboard	Foam Board
Time (ms)	273	360	660

**Table 5:** R-square and numbers of points of different methods.

	Method A		Method B		Method 12	
	R-Square	No. of Points	R-Square	No. of Points	R-Square	No. of Points
**Terrazzo**	0.9889	132	0.9902	132/12	0.9831	12
**Plasterboard**	0.9946	102	0.9967	102/12	0.9928	12
**Foam board**	0.9838	132	0.9848	132/12	0.9742	12

**Table 6:** Errors obtained by applying the proposed reflection model.

	Proposed Model Error (%)			Phong Model Error (%)		
Angle of Incidence	Terrazzo	Plasterboard	Foam Board	Terrazzo	Plasterboard	Foam Board
10	1.98	0.28	3.62	1.57	0.70	0.50
20	3.92	1.85	1.60	1.61	2.34	2.08
30	5.16	2.29	0.09	5.33	1.85	6.43
40	2.86	2.50	5.85	8.04	0.57	19.8
50	2.66	0.58	4.70	14.6	4.73	32.7
60	2.14	0.16	3.88	45.3	6.40	68.8

**Table 7:** Comparison of different light reflection models for 3 materials (obtained from the data given in [17]).

	Error (%)		
Models	Chrome	Veneer	Paint
He–Torrance	40	4.9 (2.6)	14.6
Beckmann–Spizzichino	40	N.A.*	N.A.*
Torrance–Sparrow	40	22 (7.2)	14.6
Nayar	40	7	14.6
Schlick	40 (30)	21	N.A.*

* Not tested because it is not a conductor material.
